# SOCE as a regulator of neuronal activity

**DOI:** 10.1113/JP283826

**Published:** 2023-04-22

**Authors:** Raphael Courjaret, Murali Prakriya, Khaled Machaca

**Affiliations:** 1 Calcium Signaling Group, Research Department, Weill Cornell Medicine Qatar, Qatar Foundation, Doha, Qatar; 2 Department of Physiology and Biophysics, Weill Cornell Medicine, New York, New York, USA; 3 Department of Pharmacology, Northwestern University, Feinberg School of Medicine, Chicago, Illinois, USA

**Keywords:** calcium signalling, neuronal activity, SOCE

## Abstract

Store operated Ca^2+^ entry (SOCE) is a ubiquitous signalling module with established roles in the immune system, secretion and muscle development. Recent evidence supports a complex role for SOCE in the nervous system. In this review we present an update of the current knowledge on SOCE function in the brain with a focus on its role as a regulator of brain activity and excitability

## Graphical Abstract

**Abstract figure legend** Store-operated Ca^2+^ entry affects neuronal activity by modulating several key elements of the neuronal network such as the cell body and the synaptic area. All elements of the tripartite synapse have been shown to be regulated by SOCE: pre- and post-synaptic sites as well as astrocytes that enwrap the synapse. The complex effect of SOCE between all those regulatory sites will result in a fine-tuning mechanism of neuronal excitability.

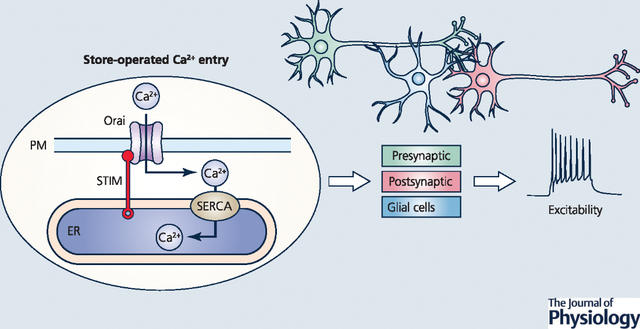

## Introduction

**Figure 1.**
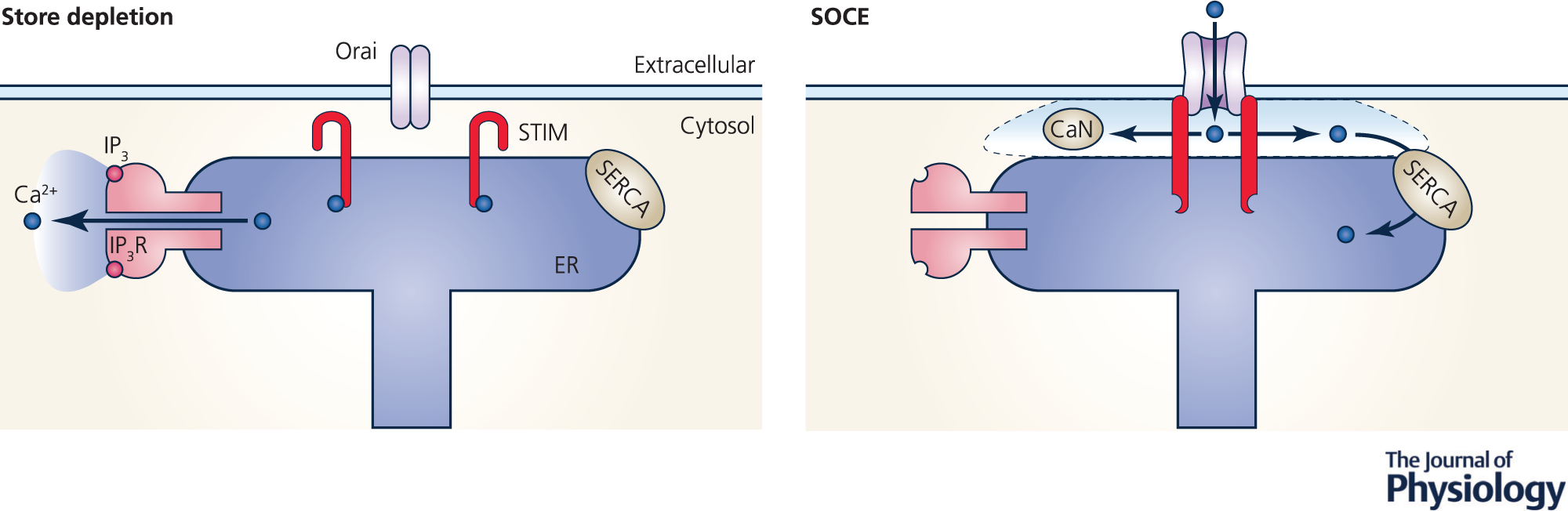
Mechanism of store operated Ca^2+^ entry Activation of IP_3_ receptors (IP_3_R) depletes the ER Ca^2+^ stores and deprives the STIM proteins from Ca^2+^, inducing their conformational change and clustering at ER–PM contact sites. Activated STIM1 recruits Orai1 to ER–PM contact sites through diffusional trapping and gates it open, allowing inward flow of extracellular Ca^2+^. The SOCE microdomain (dashed line) is spatially restricted but does encompass localized effectors such as calcineurin (CaN).

Store-operated Ca^2+^ entry (SOCE; Fig. 1) is a mechanism that links the two main Ca^2+^ sources underlying cytoplasmic Ca^2+^ signals in the cell: the extracellular space and the primary intracellular Ca^2+^ store, the endoplasmic reticulum (ER). Following agonist-dependent release of Ca^2+^ from the ER, SOCE allows for the replenishment of depleted intracellular stores and is consequently a key player in Ca^2+^ homeostasis (Schulte & Blum, 2022). In addition to this house-keeping function, SOCE is central as a signalling module for a multitude of physiological functions.

The two core elements of SOCE have now been well described: the first are stromal interaction molecule (STIM) family members, which are integral ER membrane proteins that sense the lowering of lumenal ER Ca^2+^ levels and translocate to ER–plasma membrane (PM) contact sites where they cluster and recruit the second SOCE component, the PM Ca^2+^-permeable Orai channel family members that mediate Ca^2+^ influx into the cell (Emrich et al., 2022; Lewis, 2020; Prakriya & Lewis, 2015; Putney, 2017). The SOCE machinery is not restricted to a simple STIM/Orai interaction but involves a network of proteins constituting a complex interactome that includes soluble and membrane proteins that regulate SOCE (Berlansky et al., 2021; Tedeschi et al., 2021; Wang & Demaurex, 2021).

Ca^2+^ entering the cell through SOCE activates Ca^2+^-dependent targets either in the immediate vicinity of the SOCE cluster or more distal effectors through Ca^2+^ tunnelling (Courjaret & Machaca, 2020). Effectors that localize close to the SOCE cluster, within the so-called SOCE microdomain include calcineurin, which activates the nuclear factor of activated T cells (NFAT) cascade, the archetype of SOCE signalling pathways (Hogan, 2015; Vaeth & Feske, 2018). Ca^2+^ tunnelling involves the uptake of Ca^2+^ by sarco/endoplasmic reticulum Ca^2+^-activated ATPase (SERCA) pumps near SOCE clusters, Ca^2+^ diffusion within the ER lumen, and its release through distal inositol trisphosphate (IP_3_) receptors to activate effectors (Courjaret & Machaca, 2020). Ca^2+^ tunnelling activates targets that localize quite distally from SOCE clusters in specialized cells such are pancreatic acinar cells and frog oocytes, and more recently it was shown to play a more ubiquitous function as a cortical expander of the SOCE signal to activate effectors such as Ca^2+^-activated K^+^ and Cl^−^ channels (Courjaret & Machaca, 2020; Courjaret et al., 2018; Petersen et al., 2017).

Historically, study of the function of SOCE has been largely restricted to non-excitable cells. The central argument is that excitable cells are equipped with a plethora of Ca^2+^-permeable channels at the PM that could refill stores and support multiple forms of Ca^2+^ signalling. A second reason is more technical: the cytosolic SOCE signal (*i.e.* the transient cytosolic Ca^2+^ rise induced by the opening of Orai channels) is small and easily buried in other Ca^2+^ signals triggered by action potential firing for instance. In excitable cells the recording, isolation and analysis of those Ca^2+^ signals are consequently more difficult than in non-excitable cells. Today SOCE has been clearly identified in multiple excitable tissues, including smooth muscle, skeletal muscle and heart (Emrich et al., 2022). In the brain, even the very existence of a SOCE mechanism has been heavily debated (Lu & Fivaz, 2016). However, it has become clear that SOCE and the proteins associated with it are expressed and functional in neurons and glial cells (Bollimuntha et al., 2018; Moccia et al., 2015). In addition, modifications of SOCE and Ca^2+^ homeostasis have been associated with multiple perturbations of the nervous system such as Alzheimer disease (AD), Huntington disease (HD) and chronic pain (Bollimuntha et al., 2018; Popugaeva et al., 2015; Popugaeva et al., 2017; Tsujikawa et al., 2023; Wegierski & Kuznicki, 2018; Zhang & Hu, 2020).

Despite these findings there is lack of consensus regarding a common function of SOCE in the brain with conflicting data from different cell types, brain regions, animal models and patients. This may be due at least in part to the complexity and highly networked nature of the CNS. Central to brain function is the fine tuning of neuronal excitability and activity, whether the regulation comes from within the neuron itself (changes in intrinsic properties) or from its environment through synaptic and non-synaptic contacts. Various aspects of SOCE regulation in the brain have been addressed elsewhere so will not be re-iterated here (Moccia et al., 2015; Pascual-Caro et al., 2018; Popugaeva et al., 2015; Popugaeva et al., 2017; Serwach & Gruszczynska-Biegala, 2020; Toth et al., 2016; Wegierski & Kuznicki, 2018; Zhang & Hu, 2020). Rather, in this short review we focus on neuronal excitability/activity to evaluate the contribution of SOCE.

## Expression of SOCE proteins in the nervous tissue

All five core components of the SOCE system have been identified in the brain of humans and rodents (STIM1–2, Orai1–3), using *in situ* hybridization and transcriptomics data, as summarized in various databases such as the Human Protein Atlas (https://www.proteinatlas.org/) (Sjostedt et al., 2020) and the Allen Brain Atlas (https://portal.brain-map.org/) (Lein et al., 2007). Analysis of their expression patterns throughout the brain reveals that STIM1 and STIM2 are expressed in all regions of the mouse and human brain (Table 1) with STIM1 being represented at a higher level in most brain structures. More striking, and controversial, is the status of the Orai proteins since deletion or blockade of Orai1 function causes profound changes in brain function (Dou et al., 2018; Maneshi et al., 2020; Tshuva et al., 2017), a pattern seemingly in conflict with its relatively low expression in the brain (Hartmann et al., 2021; Prakriya, 2021). Orai1 is expressed throughout the brain at low levels compared to Orai2 and Orai3 (Table 1) ranging from barely detected (Allen Brain Atlas) to one-fifth of the values obtained for Orai2 (Protein Atlas). Although important, this information has to be considered carefully in light of several factors: (1) the data do not discriminate between neurons and glial cells; (2) Orai1 function does not require a high number of proteins at the PM (Shen et al., 2021); (3) localization to subcellular domains may be critical for Orai1’s contribution to brain physiology; and (4) mRNA detection and quantification do not necessary reflect protein expression levels at a given time (Erdogmus et al., 2022).

The actual expression of the protein detected using immunocytochemistry provides additional information for the specific expression and localization of SOCE proteins in the brain. The production of reliable antibodies for Orai1 detection has been particularly challenging (Lin et al., 2013). However, several teams have been able to detect Orai1/2 and STIM1/2 in the nervous system and in many cases to confirm the validity of this detection using knock-out animals (Maneshi et al., 2020; Ryu et al., 2017; Stegner et al., 2019)

Orai1 protein has been detected in dorsal root ganglia (DRG) and hippocampal neurons in culture. *In situ*, a large screening study evaluated the expression of Orai1 in the brain of rats, humans and cynomolgus monkeys, and revealed the presence of the protein in nearly all structures (Guzman et al., 2014). Orai1 expression has been visualized in the soma and processes of cortical and hippocampal neurons (Maneshi et al., 2020; Secondo et al., 2019), and more precise image analysis was able to localize the protein in the dendritic spines in the hippocampus *in vitro* and *in situ* (Basnayake et al., 2021; Korkotian et al., 2017). However, these data need to be interpreted with caution given the technical difficulties in detecting low endogenous levels of Orai1 protein expression with currently available antibodies.

Orai2 protein expression has been reported in DRG in culture and in the hippocampus in the dentate gyrus as well as in the CA1/CA3 regions, but its subcellular localization remains to be evaluated (Stegner et al., 2019; Wei et al., 2017). Both STIM1 and STIM2 are expressed in cultured hippocampal and cortical neurons where their relative localization appear to be developmentally regulated, with STIM2 being more localized in the mature spines in hippocampal dendrites (Gruszczynska-Biegala et al., 2011; Kushnireva et al., 2020; Sun et al., 2014). STIM1 and STIM2 are also detected in the hippocampus *in situ*, with STIM1 localizing primarily to the cell body and dendrites of pyramidal cells, a pattern of expression that is also found in layer V cortical neurons (Klejman et al., 2009; Steinbeck et al., 2011). In the cerebellum, STIM1 expression has been identified in the molecular layer and in Purkinje cells, the latter being consistently reported as the highest STIM1 expressor in the cerebellum, consistent with the key role of STIM1 in the physiology of Purkinje neurons revealed by the selective deletion of the protein (Klejman et al., 2009; Ryu et al., 2017; Skibinska-Kijek et al., 2009).

Together the expression pattern of SOCE proteins in the brain measured either at the mRNA level or by immunohistochemistry is an indicator of the potential diversity and complexity of the function of the different partners in brain physiology. The different STIM and Orai isoforms vary greatly in their relative expression intensity and localization and potentially change during brain development and plasticity.

## SOCE contribution to activity of the cerebellar and hippocampal neuronal networks

The role of SOCE in brain physiology has been investigated in multiple regions of the CNS such as the spinal cord, substantia nigra, cerebellar cortex and hippocampus (Zhang & Hu, 2020). Here we focus on the cerebellum and hippocampus since this is where the most detailed information has been obtained, including synaptic transmission, neuronal plasticity and behavioural consequences of SOCE modifications.

## Hippocampus

The contribution of SOCE to the function of hippocampal neurons has been well established in acute brain slices from mice (Chen-Engerer et al., 2019; Garcia-Alvarez et al., 2015; Maneshi et al., 2020) as well as in isolated neurons in culture (Baba et al., 2003; Hori et al., 2020; Maneshi et al., 2020; Ramesh et al., 2021; Samtleben et al., 2015). The expression of STIM1–2 as well as Orai1–3 has been documented using transcriptomics and immunohistochemistry (Guzman et al., 2014; Klejman et al., 2009; Lein et al., 2007; Sjostedt et al., 2020). Although a ‘classical’ role of SOCE in replenishing ER stores has been well established (Chen-Engerer et al., 2019; Samtleben et al., 2015), the role of SOCE extends largely beyond this housekeeping function in the hippocampus. Orai1 has been argued to affect dendritic spine morphology. Immature dendritic spines adopt a filipodia shape, and when they mature into excitatory synapses, they adopt a more mushroom-like shape (Berry & Nedivi, 2017). Orai1 localizes to dendritic spines of hippocampal neurons both in culture and *in situ* (Basnayake et al., 2021; Korkotian et al., 2017). In cultured neurons the absence of Orai1 results in spines with the immature filopodia morphology (Korkotian et al., 2017). In contrast, in Orai1-knockout animals there was no visible change in the spine morphology in brain slices, mirroring the fact that resting synaptic activity was not affected in the absence of Orai1 (Maneshi et al., 2020). However, Orai1 is required for the function of mushroom spines, as its absence leads to a lower synaptic connectivity and the inability of the synapse to efficiently support synaptic plasticity during stimulation (Korkotian et al., 2017; Maneshi et al., 2020).

Additional results suggest that Orai1 has little influence on spine morphology in mature hippocampal neurons and that this function is more probably carried by Orai2 (Zhang et al., 2016). Spine stimulation with glutamate induces a transient depletion of the ER stores coupled, after a very short delay (≈100 ms), with the activation of Orai1 channels to replenish the stores, suggesting a depletion/SOCE coupling mechanism that is quicker than in non-excitable cells (Maneshi et al, 2020). In addition, the glutamate-induced Ca^2+^ signals recorded in dendritic spines were strongly impaired in *Orai1*^*fl/fl Nes-Cre*^ and *Orai1*^*fl/fl CaMKIIa-cre*^ animals, suggesting a central role for Orai1 in controlling calcium influx during excitatory synaptic transmission (Maneshi et al., 2020).

Analysis of the neuronal network activity in culture and in acute slices following either Orai1 or STIM2 silencing or overexpression did not reveal any major alterations in basal synaptic activity (Majewski et al., 2020; Maneshi et al., 2020) although some reduction in the miniature (m)EPSCs has been reported (Korkotian et al., 2017). In addition, testing for presynaptic short-term plasticity using a paired-pulsed facilitation protocol did not reveal any consequences of Orai1 alterations (Majewski et al., 2020; Maneshi et al., 2020). Conversely, recording of the long-term potentiation (LTP) at the Schaffer collateral–CA1 synapse during genetic or pharmacological (BTP-2) inhibition of Orai1 strongly reduced LTP while the afferent signal remained unaffected (Maneshi et al., 2020). Inducing ‘chemical’ LTP using an NMDA-enhancing medium indicated as well that Orai1 expression was required to generate LTP in the hippocampus (Korkotian et al., 2017; Tshuva et al., 2017).

Overexpression of Orai1 and STIM2 in neurons induced only modest changes in behaviour in the mice and did not induce premature death or neurodegeneration (Majewski et al., 2020). The behavioural consequences of the elimination of Orai1 in nestin-positive cells (neurons and glial cells) has also been carefully evaluated and revealed deficient short-term memory (as measured using a Y-maze test) and impaired learning ability (detected using a fear conditioning test) while retaining normal locomotor activity. Those defects could be attributed to an impairment of excitatory neurotransmission since they could be reproduced using the selective deletion of Orai1 in excitatory (*Orai1*^*fl/fl CaMKIIa-cre*^) but not in inhibitory neurons (*Orai1*^*fl/fl GAD2-cre*^) (Maneshi et al., 2020).

STIM1 is expressed in the ER of hippocampal neurons with a predominant localization in the soma and dendrites (Klejman et al., 2009). In those neurons, a complex mechanism has been described in which STIM1 clusters following NMDA receptor activation of a Ca^2+^-induced Ca^2+^ release mechanism. STIM1 is then responsible for the growth of ER spine content, the inhibition of L-type Ca^2^ channels and the downstream reduction of NFATc3 activation (Dittmer et al., 2017).

The overexpression of STIM1 in hippocampal neurons had no influence on basal synaptic transmission or short-term presynaptic plasticity as measured using a paired pulsed facilitation protocol or LTP but induced a strong deficit in long-term depression (Majewski et al., 2017). Conversely, and in opposition to Orai1, knocking down, STIM1 reduces basal synaptic transmission frequency and amplitude (Chanaday et al., 2021). No effect of overexpression or inhibition of STIM1 on locomotion or agility has been observed. Mice overexpressing STIM1 displayed decreased anxiety and modifications in contextual learning while the conditional deletion of STIM1 in the forebrain induced only minor learning disabilities as assessed using the Morris water maze (Garcia-Alvarez et al., 2015; Majewski et al., 2017).

Collectively these results argue that SOCE plays a role in regulating neuronal activity and plasticity with disparate specific local effects based on the cell type and subcellular location.

In addition to STIM1, STIM2 plays an important role in hippocampal function (Sun et al., 2014). Adding to the complexity of the system, STIM1 and STIM2 coexist in the same cells, and it has been suggested that they serve different functions during neuronal maturation, STIM1 being more prevalent in ‘younger’ neurons where it is associated with Ca^2+^ sparks and the generation of filopodia, while STIM2 would serve as a more ‘classical’ ER Ca^2+^ sensor to regulate Ca^2+^ stores in mature neurons (Kushnireva et al., 2020). STIM2 has been detected in synaptosomal lysates and in dendritic spines where it interacts with Orai1 and Orai2 (Korkotian et al., 2017; Zhang et al., 2016) and STIM2 inactivation induces a small increase in basal mEPSC amplitude with no changes in their frequency (Chanaday et al., 2021). In the hippocampal slice, partial store depletion using thapsigargin promotes an STIM2-dependent increase in mEPSC frequency with no alteration in presynaptic facilitation (Chanaday et al., 2021). Still, STIM2 has also been shown to be enriched in excitatory presynaptic terminals where one of its functions would be, in response to ER stress, to allow a sufficient increase in cytosolic Ca^2+^ that would in turn regulate synaptotagmin-7 and consequently facilitate synaptic transmission (Chanaday et al., 2021). The exact function of presynaptic Ca^2+^ ER stores and SOCE in the regulation of neurotransmitter release is a complex and growing area of study (Bezprozvanny & Kavalali, 2020).

The overexpression of STIM2 is also able to rescue spine aberrations in mice models of AD where a decreased STIM2 expression translates into a reduction in SOCE and in downstream CaMKII activity (Popugaeva et al., 2015; Popugaeva et al., 2017; Sun et al., 2014). STIM2 inactivation in the forebrain did not induce any alteration in locomotion, learning or memory and it required silencing of both STIM1 and STIM2 to register some behavioural changes (Garcia-Alvarez et al., 2015). Collectively these finding argue for a role for Orai1, STIM1 and STIM2 in hippocampal function. While STIM1 and STIM2 contribute to basal synaptic transmission, their main function appears to be the regulation of dendritic spine morphology, postsynaptic signalling and plasticity. As such their inactivation results mainly in learning deficiencies in the animals.

## Cerebellum

Store-depletion induced by SERCA inhibition has been shown to trigger SOCE in Purkinje and granule cells in culture (Dhanya & Hasan, 2021b; Singaravelu et al., 2008). All proteins from the SOCE ‘core’ toolkit have been identified in the cerebellum. STIM1 is expressed mostly in the cell body and in the massive dendritic tree of the Purkinje cells as well as in the granular layer, while STIM2 is expressed in the molecular and granular layers and in Purkinje cells at lower levels than STIM1 (Hartmann et al., 2014; Klejman et al., 2009; Sjostedt et al., 2020). Orai1 has been detected using immunohistochemistry in the cerebellar cortex of rats, mice and cynomolgus monkeys (Guzman et al., 2014), a result not yet confirmed since transcriptomics indicate very low expression of Orai1 as compared with Orai2 and 3 (Table 1) (Dhanya & Hasan, 2021b; Lein et al., 2007). Furthermore, large-scale immunohistochemistry failed to detect Orai1 in the cerebellum but identified Orai2 in the granular layer and Orai3 in all three layers of the cerebellar cortex (Sjostedt et al., 2020). Those findings also need to be further validated since the detection pattern at the single cell level does not clearly indicate a membrane protein.

The consequences of the selective deletion of STIM1 in Purkinje cells (STIM1^PKO^) has been particularly carefully dissected, in those cells the dendritic tree shows an altered morphology (reduced branching pattern and volume) and SOCE is reduced but not abolished, suggesting the contribution of other elements (Dhanya & Hasan, 2021b). The seminal work of Hartmann et al. (2014) revealed that basal excitatory synaptic transmission and paired pulsed facilitation were unaffected by the removal of STIM1 in the postsynaptic neuron, but that slower mGluR1-dependent synaptic events as well as IP_3_-dependent ER Ca^2+^ release were severely impaired. Further studies have indicated that although long-term synaptic plasticity was not impaired by the inactivation of STIM1 in Purkinje cells, several intrinsic factors were affected. The clearance of somatic but not dendritic cytosolic Ca^2+^ during action potential firing was delayed, leading to Ca^2+^ accumulation and in parallel to a lower firing rate and a reduced excitability. An intrinsic plasticity mechanism in which LTP induced by parallel fibre stimulation increased action potential firing was also abolished in STIM1^PKO^ animals (Dhanya & Hasan, 2021b; Ryu et al., 2017). Analysis of gene expression over time in STIM1^PKO^ animals indicated an age-dependent (animals ages 1 year *vs*. 14 weeks) regulation. Elements of the Ca^2+^ machinery such as Orai3, IP_3_R1 and calmodulin are downregulated in older animals as well as pumps and channels at the PM and genes implicated in synaptic signalling and neuronal development, indicating that age could be a crucial factor in analysing SOCE function (Dhanya & Hasan, 2021b).

Basal locomotion was not affected in the STIM1^PKO^ animals, but they exhibited deficient motor coordination as measured by a high beam test as well as poor motor learning during a rotarod protocol (Dhanya & Hasan, 2021b; Hartmann et al., 2014). When tested for the vestibulo-ocular reflex (requiring coordination of the eyes and head movement), STIM1^PKO^ mice performed well in the short term but displayed impaired memory consolidation over time (Ryu et al., 2017). While a global abnormal growth of the cell was not detected in the STIM1^PKO^ mice, a reduction in the branching pattern and in the volume of the dendritic tree of the Purkinje cell has been described, as well as an increased innervation by climbing fibres, potentially to compensate for decreased synaptic efficiency (Dhanya & Hasan, 2021b). Interestingly the defects in gene expression, synaptic connection and motor learning observed in the STIM1^PKO^ animals can be rescued following deletion of the filament-forming protein Septin 7 through an as a yet unclear mechanism (Dhanya & Hasan, 2021a).

Study of the cerebellum and particularly the Purkinje cells expands our vision of the diversity of the function of SOCE in neurons. It appears that the inactivation of STIM1 affects some intrinsic properties of the neuron, including excitability, and that SOCE affects the expression of a multitude of genes in an age-dependent fashion.

## Glia cells and SOCE

With glial cells being largely non-excitable, SOCE is likely to contribute significantly to intracellular Ca^2+^ signalling and store-refilling. Surprisingly, SOCE’s contribution to the physiology of glial cells has not yet been widely studied and remains a very interesting field of investigation. The SOCE signal (Ca^2+^ entry following store depletion) has been identified in cerebellar, hippocampal, cortical and white matter astrocytes (Kwon et al., 2017; Papanikolaou et al., 2017; Singaravelu et al., 2006; Toth et al., 2019), as well as microglia (Gilbert et al., 2016; Zhang & Hu, 2020) and oligodendrocytes ((Papanikolaou et al., 2017; Rui et al., 2020). In mouse microglia, a recent study has shown that Orai1-mediated SOCE drives inflammatory cytokine production and microglial proliferation following stimulation with proalgesic molecules or *in vivo* after nerve injury. Conditional deletion or pharmacological Orai1 inhibition was also able to alleviate allodynia in male but not female mice, indicating a sexual dimorphism in the regulation of pain sensation by microglial SOCE (Tsujikawa et al., 2023).

At the molecular level astrocytic SOCE is largely carried by STIM1/Orai1 and Orai3 complexes (Kwon et al., 2017; Toth et al., 2019). Due to their non-excitable nature, glial cells will largely rely on Ca^2+^ signalling for information processing, and spatio-temporal distribution of information (Lia et al., 2021; Lim et al., 2021). Furthermore, the precise function of SOCE in the different glial cell types remains an open question.

In the case of astrocytes, the role of SOCE can be envisaged in the context of the tripartite synapse model. The synapse is not limited to a pre- and a postsynaptic element but also includes astrocytic processes that contribute to the fine tuning of synaptic transmission (Durkee & Araque, 2019). In the hippocampus, astrocytic SOCE stimulation [through artificial store depletion or G protein-coupled receptor (GPCR) activation] potentiates gliotransmitter release through an Orai1-dependent mechanism (Toth et al., 2019). The consequence at the synapse is an increase in the frequency of IPSCs received by pyramidal neurons. The suppression of Orai1 eliminates this effect while resting synaptic activity is largely unaffected (Toth et al., 2019). At the whole animal level, the suppression of STIM1 in astrocytes decreases intracellular Ca^2+^ levels, lowers their ability to respond to adrenergic stimulation and perturbates mice sleep homeostasis (Ingiosi et al., 2020). Defining the function of SOCE in astrocytes remains an active field of investigation, with future studies aiming to understand the differential contribution of SOCE to glial physiology in different brain areas, in specialized glia, and during brain disorders whether acute or chronic.

## SOCE as a regulator of neuronal excitability

Based on the above discussion, the role of SOCE in the brain globally is likely to be complex with cell type-specific contributions. What is clear though is that SOCE does modulate neuronal physiology at both pre- and post-synaptic levels, in excitatory and inhibitory neurons and in glial cells. However, a growing body of evidence now suggests that SOCE is potentially a regulator of neuronal network excitability.

Orai1 knockout at the level of the whole brain or specifically in inhibitory interneurons increases the sensitivity of mice to chemoconvulsants (Hori et al., 2020). We observed a similar phenotype in a novel STIM1 hypomorph mouse model (Yu et al., 2022) that displays increased susceptibility to seizures in response to chemoconvulsants (our unpublished data). This argues that this susceptibility is SOCE-dependent as it is observed in both Orai1 and STIM1 loss-of-function animals. The SOCE hypomorph strain has reduced SOCE (~70% reduction) due to expression of a mutant STIM1 with an extend C-terminus that decreases its ability to concentrate at ER–PM contact sites and thus induce SOCE. The STIM1 hypomorph exhibits cardiovascular defects, primarily hypertension and tachycardia. The hypertension is due to the tachycardia which is not heart specific as it does not manifest in a heart-specific SOCE hypomorph strain but only in the global STIM1 hypomorph. We further observe an increase in sympathetic autonomic nervous system activity as well as high basal levels of circulating catecholamines in the global SOCE hypomorph, which could contribute to tachycardia (Yu et al., 2022).

Surprisingly, overexpression of Orai1 induces spontaneous seizures in adult female mice (Maciag et al., 2019) and alters the expression of epilepsy-associated genes (Majewski et al., 2019). Pharmacological inhibition of SOCE (ML-9: 1-(5-chloronaphthalenesulfonyl) homopiperazine hydrochloride or 2-APB: 2-aminoethoxydiphenyl borate) in cultured cortical neurons grown on a multi-electrode array reduces the activity of the neuronal network. The effects of both inhibitors were complex: ML-9 increased synchrony while 2-APB tended to reduce it (Steinbeck et al., 2011). In organotypic slices of the hippocampus where epileptiform activity was pharmacologically induced, those inhibitors increased bursting activity and ‘rhythmized’ the epileptic activity. The same authors also reported increased expression of STIM1 and 2 in a chronic epileptic mice model (Steinbeck et al., 2011). Finally, a recent study indicated that inactivation of the STIM2b isoform zebrafish larvae increased neuronal activity and induced a higher susceptibility to seizures when induced with a low dose of pentyleneterazole (Wasilewska et al., 2020).

As discussed above Orai1 is important in amplifying postsynaptic dendritic spine Ca2+ signals and consequently regulate LTP and learning and memory (Maneshi et al., 2020). The effects of reduced SOCE in excitatory neurons would lead to reduced neuronal activity in these neurons and thus an expected reduction in seizure threshold, contrary to what is observed in whole-brain SOCE loss-of-function animals. The global excitability of the neuronal network will therefore depend on a tight balance between the importance of SOCE in glutamatergic input to inhibitory interneurons and excitatory neurons. This argues that in terms of regulating sensitivity to chemoconvulsants, SOCE’s reduction of inhibitory interneuron excitability plays the predominant role.

Store depletion has been shown to increase neuronal excitability in DRG neurons (Wei et al., 2017) and in dorsal horn neurons of the mouse spinal cord (Dou et al., 2018) but reduces it in hippocampal pyramidal neurons (Narayanan et al., 2010). In the case of the dorsal horn neurons the authors indicate that store depletion lowers the rheobase, increases spike frequency and reduces A-type potassium currents. The proposed mechanism involved the activation of protein kinase C by Ca^2+^ entering the cell through SOCE that in turn phosphorylates the extracellular regulated kinase (ERK). Downstream, ERK downregulates A-type channels resulting in increased neuronal excitability (Dou et al., 2018). This mechanism has been linked to the reduced nociception observed in Orai1^−/−^ animals, making those channels an interesting target for the management of pathological pain (Mei et al., 2018).

As mentioned above, the deletion of STIM1 in Purkinje neurons increases excitability in a complex manner. The rheobase was unaltered while the membrane resistance as well as spike frequency were reduced; the authors suggested a complex mechanism that involves potentially several membrane channels, including gK_Ca_, and SERCA activity (Ryu et al., 2017). In hypothalamic kisspeptin neurons the rheobase was unchanged following STIM1 inactivation (Qiu et al., 2021) and similarly, in dorsal horn neurons, the silencing of Orai1 did not affect the excitability of the cell at rest (Dou et al., 2018). Interestingly, the changes in excitability induced by STIM1 inactivation could follow an age-dependent mechanism. In the old (1 year) STIM1^PKO^ animal, the Na^+^/K^+^ pump *Atp1a3*, the Ca^2+^ channel subunit *Cacng5* and the K^+^ channel-associated subunit *Kctd17* are downregulated, suggesting changes in excitability in older animals that do not exist in younger animals (17 weeks), a pattern that needs to be studied in detail (Dhanya & Hasan, 2021b). Together, the work performed on a diverse set of neuronal structures indicates that SOCE is a strong candidate for the modulation of neuronal excitability, whether it is from the modulation of the synaptic input or as changes in the intrinsic properties of the cell. The fact that those changes may be age-related is of particular interest in understanding how SOCE contributes to brain physiology in healthy and disease conditions but also during ageing.

## SOCE in neurodegenerative diseases

Considering the central role played by Ca^2+^ in neuronal and glial physiology, it is clear that perturbations in SOCE will affect multiple neurological disorders including neuropathic pain (Tsujikawa et al., 2023) and stroke (Bollimuntha et al., 2017). This is also the case for several neurodegenerative diseases such as ataxia, AD, HD and Parkinson disease (Bollimuntha et al., 2018; Pchitskaya et al., 2018; Popugaeva et al., 2015; Popugaeva et al., 2017; Secondo et al., 2018; Wegierski & Kuznicki, 2018; Zhang & Hu, 2020). In the case of AD, the dysregulation of SOCE and Ca^2+^ homeostasis in astrocytes (Linde et al., 2011) and in neurons at both pre- (Deng et al., 2020; Lerdkrai et al., 2018) and importantly at postsynaptic sites (Sun et al., 2014) is now considered a critical element in the development of the disease. The contribution of alterations in Ca^2+^ signalling to synaptic loss in AD has been particularly well evaluated. A proposed mechanism to explain the elimination of mushroom spines in AD involves an overload of Ca^2+^ in the ER and a compensation loop that would then promote a reduction in SOCE through the downregulation of STIM2. Downstream this perturbation would destabilize the crucial and tightly regulated phosphorylation/dephosphorylation balance governed by two calmodulin-sensitive enzymes involved in the stability of the spines: CaMKII and calcineurin (Popugaeva et al., 2017). The development of a positive SOCE modulator to mitigate the development of AD is consequently an attractive area of research (Popugaeva et al., 2015).

In the case of HD, a different mechanism emerges. Perturbations in Ca^2+^ homeostasis have been observed in neurons derived from pluripotent stem cells obtained from HD patients and in striatal medium spiny neurons (MSNs) isolated from HD mouse models. A sensitized IP_3_ receptor promotes an ER Ca^2+^ leak, increasing SOCE intensity as well as STIM2 expression, and leads to synaptic loss (Czeredys, 2020; Vigont et al., 2021; Wegierski & Kuznicki, 2018; Wu et al., 2016). The reduction in spine density observed in the YAC128 mouse model (expressing the full-length human huntingtin gene with a 128 glutamine repeat expansion in exon 1) could be rescued by suppression of several proteins involved in the SOCE pathway (STIM1, TRPC1, TRPC6, Orai1 and Orai2) and knocking out TRPC1 in the YAC128 mouse is able to improve its motor performance (Wu et al., 2018). Interestingly, a SOCE inhibitor, the neuroprotective agent EVP4593, is also able to rescue spine loss in the same mouse model, making it an interesting candidate for HD treatment (Grekhnev et al., 2022; Wu et al., 2016). The various, and sometimes opposite, alterations in intracellular Ca^2+^ signalling leading to synaptic loss illustrate the complexity and tight regulation of those mechanisms in the brain and that there is no single ‘neuronal SOCE’ but multiple modules depending on localization, function and time.

## Does SOCE support Ca^2+^ tunnelling in the brain?

The SOCE microdomain is restricted in space and time, so any effector controlled by Ca^2+^ influx, such as calcineurin (Schober et al., 2019) and adenylate cyclase 8 (Sanchez-Collado et al., 2021), has to localize within it. Ca^2+^ tunnelling expands the SOCE microdomain to distant targets (Courjaret & Machaca, 2020; Petersen et al., 2017). Ca^2+^ ions entering the cells through SOCE are pumped into the ER by SERCA and released in the vicinity of specific Ca^2+^ effectors by IP_3_ receptors primarily in the cell cortex, thus expanding the SOCE signal cortically. Although it has been described in several cell types such as acinar cells, the frog oocyte and HeLa cells, tunnelling has not yet been reported in neurons or glial cells. One of the requirements for tunnelling to occur is the localization of the ER release channel (e.g. the IP_3_ receptor) close to the target. This is the case for channels at the PM of neurons and astrocytes such as the Ca^2+^-activated chloride channel ANO1 (Jin et al., 2013; Jin et al., 2016) and the Ca^2+^-activated potassium channels BK (Shah et al., 2021; Weaver et al., 2007) as well as the mitochondria (Kushnireva & Korkotian, 2022). Another key element in the tunnelling mechanism is the central role played by the SERCA pump that contributes to restriction of the SOCE microdomain and refilling of the ER stores (Courjaret & Machaca, 2014; Courjaret & Machaca, 2020; Courjaret et al., 2018; Petersen et al., 2017). Interestingly, in the Purkinje neurons of STIM1^PKO^ mice, the reduction of excitability requires SERCA activity (Ryu et al., 2017). The polarized nature of neurons and astrocytes and the existence of highly specialized regions such as the synapse and the end-feet of astrocytes make both cell types interesting candidates to assess whether Ca^2+^ tunnelling downstream of SOCE is an important mechanism in the nervous system.

## Conclusions and perspectives

The role of SOCE in the physiology of the nervous system remains largely to be discovered. A lot is known from specific structures such as the plasticity mechanisms in the hippocampus or the Purkinje cell in the cerebellum. Multiple knock-out/knock-in strategies have been tested with often little measurable effect at the behavioural level. The full inactivation of a gene might not always be the ideal strategy to study a protein function since it can trigger multiple compensatory mechanisms and have developmental consequences that affect the mechanisms in question. Hence, the new SOCE hypormorph mouse model with 70% reduction in SOCE may provide a good model for further studies in the brain (Yu et al., 2022).

Understanding the contribution of SOCE or individual Orai/STIM proteins to the global activity of the brain will be challenging. In the hippocampus, for instance, postsynaptic Orai1 in dendritic spines contributes to the rapid Ca2+ signal and the most straightforward interpretation of the data is that Orai1 is involved in sustaining elevated levels of excitatory synaptic transmission in excitatory neurons following tetanus-like stimuli, which elicit plasticity in dendritic spines. Concurrently Orai1 is required for the regulation of excitatory neurons by inhibitory interneurons during stimulation by chemoconvulsants (Hori et al., 2020; Maneshi et al., 2020).

One common pattern that emerges is the general absence of a strong contribution of SOCE to brain basal activity. There are no obvious defects in behaviour associated with the inactivation of individual SOCE proteins. For instance, when STIM1 is inactivated in Purkinje cells, which are central for motor coordination, animals do not exhibit overt motor deficits unless challenged (Hartmann et al., 2014). In a similar way, basal synaptic transmission is not affected when Orai1 is removed from hippocampal excitatory synapses (Maneshi et al., 2020) but differences will appear when the neuronal networks are challenged either by inducing synaptic plasticity or by triggering seizures (Hori et al., 2020; Maneshi et al., 2020). The contribution of SOCE to brain physiology might therefore be conceived as a critical regulator during stress of high-demand conditions. Understanding its function will therefore require analysis of intrinsic neuronal parameters such as excitability as well as synaptic activity, connectivity and plasticity in resting conditions but also under stress. Finally, specific mechanisms in healthy and pathological brain functions might be associated with a differential contribution of SOCE to brain physiology during ageing and could represent a promising area of investigation.

## Supplementary Material

Supplem Info

## Figures and Tables

**Table 1.** RNA expression of SOCE genes in the brain

Human – *Human Protein Atlas* – nTPM										

	Cx	Hp	Ag	B. Ggl	Th	Hyp	Mid	Cb	Pons	Med

Orai1	6.8	3.8	4.4	4.6	5.1	4.9	4.9	5.2	5.5	5.5
Orai2	21.2	24.4	18.7	13.7	27.7	26.2	20	16.5	24.5	21
Orai3	17.1	15.3	18	16.5	20.5	16.3	18.5	16.9	17.1	18.6
*Ratio Orai2/1*	*3.1*	*6.4*	*4.3*	*3.0*	*5.4*	*5.3*	*4.1*	*3.2*	*4.5*	*3.8*
STIM1	34.5	30.5	32.9	31.5	26.9	23.1	22.9	18.1	18.9	17.4
STIM2	14.3	17.7	11.5	14.3	13.7	12.4	12.2	11.1	12.5	12.7
*Ratio STIM1/2*	*2.4*	*1.7*	*2.9*	*2.2*	*2.0*	*1.9*	*1.9*	*1.6*	*1.5*	*1.4*

Mouse – *Human Protein Atlas* – nTPM									

	Cx	Hp	Ag	B. Ggl	Th	Hyp	Mid	Cb	Pons+Med

Orai1	6.7	7.4	6.8	7.9	5.2	6.2	4.2	6.4	6.1
Orai2	39.7	111.6	48.3	45.9	23.7	26	22.8	28.5	16.3
Orai3	23	29.8	18.9	14.6	18.4	15.1	12.1	26.1	11.6
*Ratio Orai2/1*	*5.9*	*15.1*	*7.1*	*5.8*	*4.6*	*4.2*	*5.4*	*4.5*	*2.7*
STIM1	47.9	44.2	48.1	63.5	37.1	54.2	43.2	41	33.4
STIM2	26	58.2	24.7	28.3	16.6	18.6	20.1	15.9	14.9
*Ratio STIM1/2*	*1.8*	*0.8*	*1.9*	*2.2*	*2.2*	*2.9*	*2.1*	*2.6*	*2.2*

Mouse – *Allen Mouse Brain Atlas – ISH* – raw values								

	Cx	Hp	Th	Hyp	Mid	Cb	Pons	Med

Orail	0.1	0.11	0.06	0.02	0.09	0.11	0.12	0.11
Orai2	1.9	5.9	1.76	0.27	1.24	2.17	0.46	0.62
Orai3	0.72	0.82	0.13	0.09	0.3	1.87	0.44	0.5
*Ratio Orai 2/1*	*5.9*	*15.1*	*4.6*	*4.2*	*5.4*	*4.5*	*4.5*	*4.5*
STIMI	10.5	10.48	6.68	9.28	7.25	5.59	6.54	5.8
STIM2 11.23	12.57	3.66	2.66	3.84	3.28	2.26	3.65
*Ratio STIM1/2*	*0.9*	*0.8*	*1.8*	*3.5*	*1.9*	*1.7*	*2.9*	*1.6*

Data have been extracted from the databases indicated in the headers. The highest value between Orai or STIM proteins is highlighted in orange. Cx: cortex, Hp: hippocampus, Ag: amygdala, B.Ggl: basal ganglia, Th: thalamus, Hyp: hypothalamus, Mid: midbrain, Cb: cerebellum, Med: medulla.
