# Supplementary material for: SOCE as a regulator of neuronal activity
Source: J Physiol. Author manuscript; Available in PMC 2025 Apr 2. (PMC11963908; doi:10.1113/JP283826)
Supplement: Supplem Info [file NIHMS2060014-supplement-Supplem_Info.pdf]

The Journal of Physiology

SOCE as a regulator of neuronal activity

Raphael Courjaret, Murali Prakriya, and Khaled Machaca
DOI: 10.1113/JP283826

Corresponding author(s): Raphael Courjaret (rac2017@qatar-med.cornell.edu)

The following individual(s) involved in review of this submission have agreed to reveal their identity: Ilya Bezprozvanny (Referee #1)

Review Timeline:

Submission Date:	08-Jan-2023
Editorial Decision:	22-Feb-2023
Revision Received:	20-Mar-2023
Accepted:	28-Mar-2023

Senior Editor: Laura Bennet

Reviewing Editor: Samuel Young

Transaction Report:

(Note: With the exception of the correction of typographical or spelling errors that could be a source of ambiguity, letters and reports are not edited. Depending on transfer agreements, referee reports obtained elsewhere may or may not be included in this compilation. Referee reports are anonymous unless the Referee chooses to sign their reports.)

Dear Dr Courjaret,

Re: JP-SR-2023-283826 "SOCE as a regulator of neuronal activity" by Raphael Courjaret, Murali Prakriya, and Khaled Machaca

Thank you for submitting your manuscript to The Journal of Physiology. It has been assessed by a Reviewing Editor and by 1 expert referee and we are pleased to tell you that it is acceptable for publication following satisfactory revision.

Please advise your co-authors of this decision as soon as possible.

The referee reports are copied at the end of this email.

Please address all the points raised and incorporate all requested revisions or explain in your Response to Referees why a change has not been made. We hope you will find the comments helpful and that you will be able to return your revised manuscript within 4 weeks. If you require longer than this, please contact journal staff: jp@physoc.org.

Your revised manuscript should be submitted online using the link in your Author Tasks: Link Not Available. This link is accessible via your account as Corresponding Author; it is not available to your co-authors. If this presents a problem, please contact journal staff (jp@physoc.org). Image files from the previous version are retained on the system. Please ensure you replace or remove any files that are being revised.

If you do not wish to submit a revised version of your manuscript, you must inform our journal staff (jp@physoc.org) or reply to this email to request withdrawal. Please note that a manuscript must be formally withdrawn from the peer review process at one journal before it may be submitted to another journal.

TRANSPARENT PEER REVIEW POLICY: To improve the transparency of its peer review process, The Journal of Physiology publishes online as supporting information the peer review history of all articles accepted for publication. Readers will have access to decision letters, including Editors' comments and referee reports, for each version of the manuscript, as well as any author responses to peer review comments. Referees can decide whether or not they wish to be named on the peer review history document.

ABSTRACT FIGURES: Authors may use The Journal's premium BioRender account to create/redraw their Abstract Figures (and any other suitable schematic figures). Information on how to access this account is here: https://physoc.onlinelibrary.wiley.com/journal/14697793/biorender-access.

This will enable Authors to create and download high-resolution figures. If authors have used the free BioRender service, they can use the instructions provided in the link above to download a high-resolution version suitable for publication.
The link provided should only be used for the purposes of this submission. Authors will be charged for figures created on this account if they are not related to this manuscript submission.

LANGUAGE EDITING AND SUPPORT FOR PUBLICATION: If you would like help with English language editing, or other article preparation support, Wiley Editing Services offers expert help, including English Language Editing, as well as translation, manuscript formatting, and figure formatting at www.wileyauthors.com/eeo/preparation. You can also find resources for Preparing Your Article for general guidance about writing and preparing your manuscript at www.wileyauthors.com/eeo/prepresources.

REVISION CHECKLIST:
Upload a full Response to Referees file. To create your 'Response to Referees' copy all the reports, including any comments from the Senior and Reviewing Editors, into a Microsoft Word, or similar, file and respond to each point, using font or background colour to distinguish comments and responses and upload as the required file type.

Please upload two versions of your manuscript text: one with all relevant changes highlighted and one clean version with no changes tracked. The manuscript file should include all tables and figure legends, but each figure/graph should be uploaded as separate, high-resolution files.

You may also upload:
- 'Potential Cover Art' for consideration as the issue's cover image
- Appropriate Supporting Information (Video, audio or data set: see https://jp.msubmit.net/cgi-bin/main.plex?form_type=display_requirements#supp).

We look forward to receiving your revised submission.

If you have any queries, please reply to this email and we will be pleased to advise.

Yours sincerely,

Professor Laura Bennet
Senior Editor
The Journal of Physiology
https://jp.msubmit.net
http://jp.physoc.org
The Physiological Society
Hodgkin Huxley House
30 Farringdon Lane
London, EC1R 3AW
UK
http://www.physoc.org
http://journals.physoc.org

REQUIRED ITEMS

Please include an Abstract Figure file, as well as the figure legend text within the main article file. The Abstract Figure is a piece of artwork designed to give readers an immediate understanding of the Review Article and should summarise the main conclusions. If possible, the image should be easily 'readable' from left to right or top to bottom. It should show the physiological relevance of the Review so readers can assess the importance and content of the article. Abstract Figures should not merely recapitulate other figures in the Review. Please try to keep the diagram as simple as possible and without superfluous information that may distract from the main conclusion of the Review. Abstract Figures must be provided by authors no later than the revised manuscript stage and should be uploaded as a separate file during online submission labelled as File Type 'Abstract Figure'. Please ensure that you include the figure legend in the main article file. All Abstract Figures will be sent to a professional illustrator for redrawing and you may be asked to approve the redrawn figure before your paper is accepted.

Please upload separate high quality figure files via the submission form.

EDITOR COMMENTS

Reviewing Editor:

The reviewers found the review timely and important. Please create a separate section on the role of SOCE in neurodegeneration to be included in the revision.

REFEREE COMMENTS

Referee #1:

This is an interesting and timely review on the subject important for physiology and neuroscience. Overall it is well written but I have a few suggestions/comments that I hope will help to improve this paper and enhance its impact

1. Separate section of the role of SOCE in neurodegeneration need to be included. At the moment references to this important subject area are scattered in the text and difficult to understand impact of this work

2. Please correct typo: {Maneshi, 2020 #32)},

3. Very odd choice to cite "Popugaeva E & Bezprozvanny I. (2018). STIM proteins as regulators of neuronal store-operated calcium influx. Neurodegener Dis Manag 8, 5-7". This is a relatively minor editorial. There are several reviews on this subject written by Ilya Bezprozvanny and his colleagues, please cite these. For example:

Popugaeva E, Vlasova OL, Bezprozvanny I. Restoring calcium homeostasis to treat Alzheimer's disease: a future perspective. Neurodegener Dis Manag. 2015 Oct;5(5):395-8. doi: 10.2217/nmt.15.36. Epub 2015 Oct 19. PMID: 26477700; PMCID: PMC5558533.

Popugaeva E, Pchitskaya E, Bezprozvanny I. Dysregulation of neuronal calcium homeostasis in Alzheimer's disease - A therapeutic opportunity? Biochem Biophys Res Commun. 2017 Feb 19;483(4):998-1004. doi: 10.1016/j.bbrc.2016.09.053. Epub 2016 Sep 15. PMID: 27641664; PMCID: PMC5303663.

4. Please also cite and discuss recent review on closely related subject:

Bezprozvanny I, Kavalali ET. Presynaptic endoplasmic reticulum and neurotransmission. Cell Calcium. 2020 Jan;85:102133. doi: 10.1016/j.ceca.2019.102133. Epub 2019 Nov 27. PMID: 31812114; PMCID: PMC7971302.

END OF COMMENTS

REFEREE COMMENTS

Referee #1:

This is an interesting and timely review on the subject important for physiology and neuroscience. Overall it is well written but I have a few suggestions/comments that I hope will help to improve this paper and enhance its impact

We thank the referee for the suggestions and corrections, we incorporated them in a revised version.

1. Separate section of the role of SOCE in neurodegeneration need to be included. At the moment references to this important subject area are scattered in the text and difficult to understand impact of this work

We have added a new separate section that highlights the role of SOCE in neurodegenerative diseases.

2. Please correct typo: {Maneshi, 2020 #32)},

Corrected, there was a software bug, thanks.

3. Very odd choice to cite "Popugaeva E & Bezprozvanny I. (2018). STIM proteins as regulators of neuronal store-operated calcium influx. Neurodegener Dis Manag 8, 5-7". This is a relatively minor editorial. There are several reviews on this subject written by Ilya Bezprozvanny and his colleagues, please cite these. For example:

Popugaeva E, Vlasova OL, Bezprozvanny I. Restoring calcium homeostasis to treat Alzheimer's disease: a future perspective. Neurodegener Dis Manag. 2015 Oct;5(5):395-8. doi: 10.2217/nmt.15.36. Epub 2015 Oct 19. PMID: 26477700; PMCID: PMC5558533.

Popugaeva E, Pchitskaya E, Bezprozvanny I. Dysregulation of neuronal calcium homeostasis in Alzheimer's disease - A therapeutic opportunity? Biochem Biophys Res Commun. 2017 Feb 19;483(4):998-1004. doi: 10.1016/j.bbrc.2016.09.053. Epub 2016 Sep 15. PMID: 27641664; PMCID: PMC5303663.

The citations were updated according to the suggestions of the referee.

4. Please also cite and discuss recent review on closely related subject:
Bezprozvanny I, Kavalali ET. Presynaptic endoplasmic reticulum and neurotransmission. Cell Calcium. 2020 Jan;85:102133. doi: 10.1016/j.ceca.2019.102133. Epub 2019 Nov 27. PMID: 31812114; PMCID: PMC7971302.

This more recent review is now highlighted in the text.

Dear Dr Courjaret,

Re: JP-SR-2023-283826R1 "SOCE as a regulator of neuronal activity" by Raphael Courjaret
Murali Prakriya
Khaled Machaca

I am pleased to tell you that your Symposium Review article has been accepted for publication in The Journal of Physiology, subject to any modifications to the text that may be required by the Journal Office to conform to House rules.

IMPORTANT
Please provide a legend to accompany your abstract figure (as a Word file, please). Our typesetters will need this. Please email your legend file to us at: jp@physoc.org

NEW POLICY: In order to improve the transparency of its peer review process, The Journal of Physiology publishes online as supporting information the peer review history of all articles accepted for publication. Readers will have access to decision letters, including all Editors' comments and referee reports, for each version of the manuscript and any author responses to peer review comments. Referees can decide whether or not they wish to be named on the peer review history document.

The last Word version of the paper submitted will be used by the Production Editors to prepare your proof. When this is ready, you will receive an email containing a link to Wiley's Online Proofing System. The proof should be checked and corrected as quickly as possible.

All queries at proof stage should be sent to tjp@wiley.com.

The accepted version of the manuscript is the version that will be published online until the copy edited and typeset version is available. Authors should note that it is too late at this point to offer corrections prior to proofing. Major corrections at proof stage, such as changes to figures, will be referred to the Reviewing Editor for approval before they can be incorporated. Only minor changes, such as to style and consistency, should be made a proof stage. Changes that need to be made after proof stage will usually require a formal correction notice.

Are you on Twitter? Once your paper is online, why not share your achievement with your followers. Please tag The Journal (@jphysiol) in any tweets and we will share your accepted paper with our 22,000+ followers!

Yours sincerely,

Professor Laura Bennet
Senior Editor
The Journal of Physiology
https://jp.msubmit.net
http://jp.physoc.org
The Physiological Society
Hodgkin Huxley House
30 Farringdon Lane
London, EC1R 3AW
UK
http://www.physoc.org
http://journals.physoc.org

REQUIRED ITEMS:

Please provide a legend to accompany your abstract figure (Word file, please).

EDITOR COMMENTS:

Reviewing Editor:

The manuscript has addressed all reviewer concerns.

REFEREE COMMENTS:

Referee #1:

The paper has been adequately revised and significantly improved.

----

* IMPORTANT NOTICE ABOUT OPEN ACCESS *

To assist authors whose funding agencies mandate public access to published research findings sooner than 12 months after publication, The Journal of Physiology allows authors to pay an open access (OA) fee to have their papers made freely available immediately on publication.

You will receive an email from Wiley with details on how to register or log-in to Wiley Authors Services where you will be able to place an OnlineOpen order.

You can check if you funder or institution has a Wiley Open Access Account here: https://authorservices.wiley.com/author-resources/Journal-Authors/licensing-and-open-access/open-access/author-compliance-tool.html.

Your article will be made Open Access upon publication, or as soon as payment is received.

If you wish to put your paper on an OA website such as PMC or UKPMC or your institutional repository within 12 months of publication you must pay the open access fee, which covers the cost of publication.

OnlineOpen articles are deposited in PubMed Central (PMC) and PMC mirror sites. Authors of OnlineOpen articles are permitted to post the final, published PDF of their article on a website, institutional repository, or other free public server, immediately on publication.

Note to NIH-funded authors: The Journal of Physiology is published on PMC 12 months after publication, NIH-funded authors DO NOT NEED to pay to publish and DO NOT NEED to post their accepted papers on PMC.

1st Confidential Review **20-Mar-2023**
